# Burden of COVID-19 Mortality and Morbidity in Poland in 2020

**DOI:** 10.3390/ijerph19095432

**Published:** 2022-04-29

**Authors:** Katarzyna Orlewska, Dorota Kozieł, Justyna Klusek, Ewa Orlewska

**Affiliations:** Collegium Medicum, Jan Kochanowski University, 25-369 Kielce, Poland; dkoziel@ujk.edu.pl (D.K.); jsklusek@ujk.edu.pl (J.K.); eorl@ujk.edu.pl (E.O.)

**Keywords:** Poland, COVID-19, burden of disease, years of potential life lost, quality adjusted years of life lost

## Abstract

In 2020 COVID-19 caused 41,442 deaths in Poland. We aimed to estimate the burden of COVID-19 using years of potential life lost (YPLL) and quality-adjusted years of life lost (QALYL). YPLL were calculated by multiplying the number of deaths due to COVID-19 in the analyzed age/sex group by the residual life expectancy for that group. Standard and country-specific (local) life tables were used to calculate SPYLL and LPYLL, respectively. QALYL were calculated adjusting LPYLL due to COVID-19 death by age/sex specific utility values. Deaths from COVID-19 in Poland in 2020 caused loss of 630,027 SPYLL, 436,361 LPYLL, and 270,572 QALYL. The loss was greater among men and rose with age reaching the maximum among men aged 65–69 and among women aged 70–74. Burden of COVID-19 in terms of YPLL is proportionate to external-cause deaths and was higher than the burden of disease in the respiratory system. Differential effects by sex and age indicate important heterogeneities in the mortality effects of COVID-19 and justifies policies based not only on age, but also on sex. Comparison with YPLL due to other diseases showed that mortality from COVID-19 represents a substantial burden on both society and on individuals in Poland.

## 1. Introduction

In Poland in 2020 the difference between the real (478,878) and the expected (420,325) number of deaths amounted to 58,533 [1]. As the highest death rate was noted in the last quarter of the year, there are no doubts that the higher death rate in 2020 compared to previous years mostly comes from the COVID-19 pandemic, with the peak of new cases and deaths in the last months of 2020. According to data provided by Statistics Poland, COVID-19 caused 41,442 deaths in 2020 [2], representing about 60% of the noted increase of mortality and nearly 8% of all deaths in Poland.

The aim of this study was to assess the impact of mortality due to COVID-19 in Poland in 2020 by estimating the years of potential life lost (YPLL) and quality-adjusted years of life lost (QALYL). Furthermore, we calculated QALYL due to COVID-19 morbidity in Poland in 2020. YPLL estimates the average time an individual would have lived if he or she not died prematurely and is considered as a more comprehensive measure of the impact of mortality caused by a given disease on population health compared to the number of deaths alone [3]. QALYL considers both the life expectancy at the age of death and the age-specific utility values for individuals, reflecting the impact of mortality on population health more accurately than YPLL. A QALY decrement attributable to the time spent with COVID-19 symptoms allowed to estimate the burden of COVID-19 morbidity. Estimations of YPLL and QALYL are imperative for an understanding of the disease burden on society and in order to provide additional information which might be useful in national and international comparisons.

To our knowledge, this is the first study concerning the burden of COVID-19 in Poland. We focused on the year 2020, when COVID-19 vaccination was not available and the spread of SARS-CoV-2 was limited mainly by social distancing, use of facial masks and hand disinfection. We hope that the results of this study will contribute to other COVID-19 studies, allowing comparisons of the impact of the pandemic in Poland and other countries and regions. This study is also a starting point for later analyses, when both vaccination and virus evolution will alter the course of pandemic. Based on data from 2020 it will also be possible to determine the extent to which mortality due to other causes will have changed in the coming years.

## 2. Materials and Methods

Only deaths with documented COVID-19 as cause of death (ICD-10 code: U07.1) for the year 2020 were considered in the analysis. The source of data was a report provided by Statistics Poland [2]. Deaths due to COVID-19 were defined in line with recommendations issued by the National Institute of Public Health—National Institute of Hygiene (NIPH–NIH) [4]. A NIPH–NIH report [5] was the source of data for the number of laboratory-confirmed COVID-19 cases and number of patients hospitalized due to COVID-19 in Poland in 2020. Systematic review and meta-analysis of 95 unique studies with 29,776,306 individuals who underwent testing was used to calculate percentage of symptomatic cases in the confirmed COVID-19 population [6].

YPLL in any age group were calculated by multiplying the number of deaths due to COVID-19 in the analyzed age group by the residual life expectancy for that age group: YPLL = N_x,g_ × SLE_x,g_, where N is the number of deaths at age × among persons with sex g in a given year, and SLE is the life expectancy at age × among persons with sex g. YPLL was calculated as country-specific (local) potential years of life lost (LPYLL) and standard potential years of life lost (SPYLL). Country-specific life expectancy values for each age for men and women living in Poland was derived from life tables for 2020, with a life expectancy of 81.75 years at birth in women and 74.07 years in men [7]. SPYLL was determined by the average life expectancy at the age of death, using the up-to-date reference life tables proposed by the Institute for Health Metrics and Evaluation and recently used in the GBD 2015 study, with a normative standard life expectancy of 86.59 years at birth for both men and women [8]. The resulting SPYLL figures are more abstract, as they do not reflect mortality as it exists in Poland but can be used for international and cross-time comparisons. No cut-off for age was used for the calculation, and age at death at each 5-year interval was a midpoint of the range. The LPYLL and SPYLL were calculated with a time discount rate at 0% and no age-weighting, as recommended in the GBD study and adopted by the WHO [9].

QALYL caused by COVID-19 mortality were calculated adjusting LPYLL due to COVID-19 death by age and sex specific utility values, obtained using the European Quality of Life 5 Dimensions 3 Level Version (EQ-5D-3L) questionnaire in a large, representative sample of the Polish population [10]. To estimate the burden of COVID-19 morbidity, a QALY decrement attributable to the time spent with COVID-19 symptoms was applied to anyone experiencing a detected infection. Disutility weights were obtained from a recent report on pricing models for COVID-19 treatments published by the Institute for Clinical and Economic Review in which the disutility weights were derived from data collected in patients experiencing influenza and Clostridium difficile infection [11]. In our analysis these weights were applied for each day that a patient experienced symptoms and was hospitalized for COVID-19, dependent on their level of care. COVID-19 symptoms were assumed to persist for 14 days in all non-hospitalized patients, representing the median time from onset to clinical recovery for mild cases reported by the World Health Organization-China Joint Mission on COVID-19 [12]. Duration of symptoms before hospitalization was assumed to be 4 days [13]. For individuals hospitalized for COVID-19, a length of stay (LOS) of 12 days was assumed, representing the mean LOS among Polish patients [14]. Potential late aftermath of COVID-19 has not been considered. Absolute, mean and per 100,000 inhabitants LPYLL, SPYLL and QALYL are presented.

## 3. Results

### 3.1. Basic Epidemiologic Data

According to the NIPH–NIH report there were 1,287,745 cases of laboratory-confirmed SARS-CoV-2 in the year 2020 in Poland [5]. Based on systematic review and meta-analysis of 95 unique studies with 29,776,306 individuals who underwent testing, we assumed that 59.5% of the confirmed COVID-19 population was symptomatic [6]. 102,412 patients (8%) were hospitalized [5], among them 10% was admitted to an intensive care unit (ICU) and placed on a ventilator (calculated from Polish data as for January 2021).

In 2020, 41,442 people died due to COVID-19 (ICD-10 code: U07) in Poland. 1419 cases were diagnosed based on clinical symptoms only (virus not identified, ICD-10 code: U07.2) [2]. In consequence, 40,023 deaths with laboratory-confirmed COVID-19 diagnosis (virus identified, ICD-10 code: U07.1) were included in the analysis, 58% of which were among men and 83% were at least 65 years old. Considering both sexes separately the percentage of deceased younger than 65 was 20% among men and 12% among women.

### 3.2. Years of Life Lost Due to Death from COVID-19

The total LPYLL due to death from COVID-19 in Poland in 2020 amounted to 436,361 years, 57% of which were lost by men. Mean LPYLL was similar for men and women: 10.83 and 11.00 years per death, respectively. Per 100,000 inhabitants the loss was greater among men than women (1350.64 years vs. 938.24).

The total SPYLL attributable to deaths from COVID-19 amounted to 630,027 years, 63% of which was lost by men. Both mean SPYLL and SPYLL per 100,000 persons was higher in men (17.2 and 2143.43, respectively) than in women (13.76 and 1173.51, respectively).

**Figure 1:**
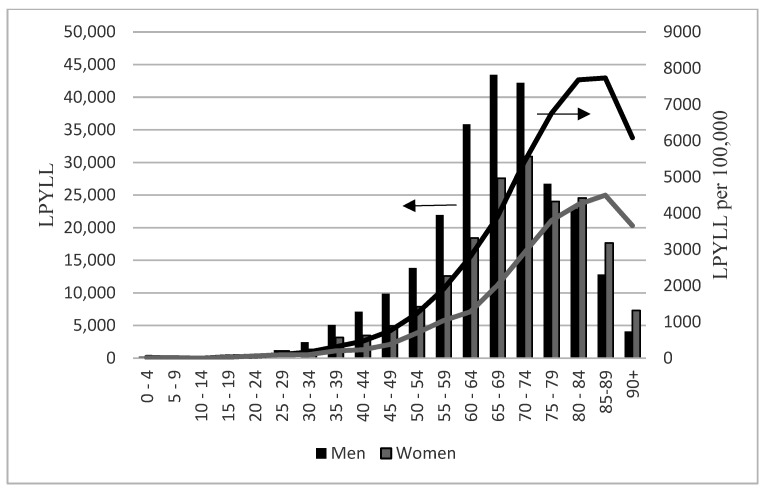
Country-specific (local) potential years of life lost (LPYLL) among people with COVID-19 in Poland in 2020 by age and sex. LPYLL—scale on the left, columns; LPYLL per 100,000—scale on the right, lines.

**Figure 2:**
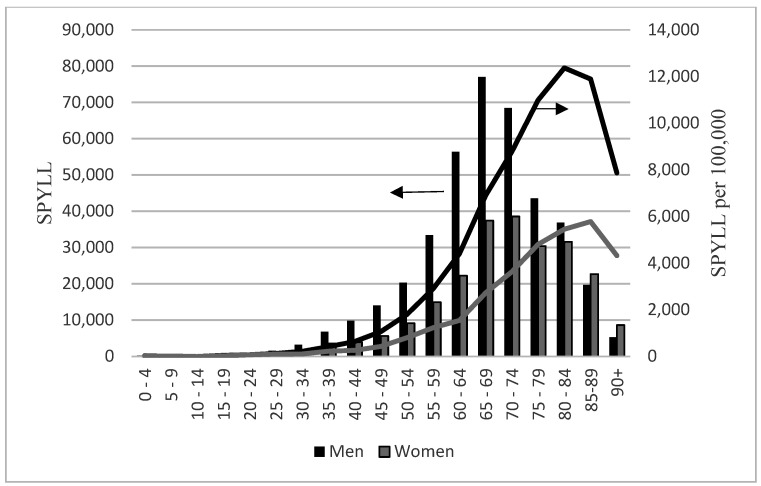
Standard potential years of life lost (SPYLL) among people with COVID-19 in Poland in 2020 by age and sex. SPYLL—scale on the left, columns; SPYLL per 100,000—scale on the right, lines.

The number of both LPYLL and SPYLL rose, with age reaching the maximum among men aged 65–69 and among women aged 70–74, to subsequently decline in advanced age (Figure 1 and Figure 2). Given that only 17% of persons who died were aged under 65 years, the share of YPLL due to COVID-19 accounted for this group is considerable: 35% of LPYLL and 33% of SPYLL with mean LPYLL and SPYLL 23 and 31.8 years, respectively. By comparison, persons older than 65 years lost an average of 8.5 LPYLL and 12.4 SPYLL due to COVID-19. Men, except for those aged 5–9 and above 80, displayed larger absolute loss of life years due to deaths from COVID-19 than women. In relative terms, the number of LPYLL and SPYLL in both sexes rose with age for persons aged 15–85 and declined for those aged 85 and more (Figure 1 and Figure 2). With the exception of persons aged 5–9, LPYLL per 100,000 and SPYLL per 100,000 were higher in men than in women.

A comparison of the SPYLL and LPYLL age distribution revealed that the effect of using loss function corresponding to longer life expectancies is more pronounced in men and in more advanced age groups. The strongest impact of prolonged life-expectancy was identified for people aged 65–69 with SPYLL being in men 1.8 times and in women 1.4 times higher than LPYLL.

### 3.3. Quality Adjusted Years of Life Lost Due to COVID-19 Mortality and Morbidity

**Figure 3:**
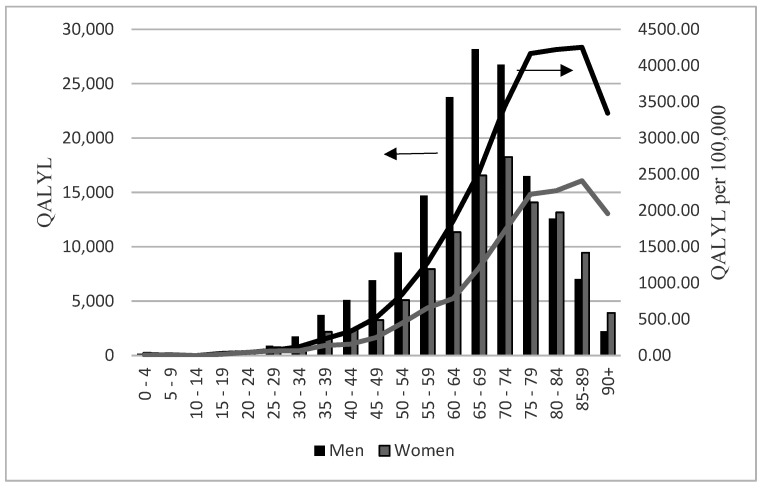
Quality adjusted years of life lost (QALYL) among people with COVID-19 in Poland in 2020 by age and sex. QALYL—scale on the left, columns; QALYL per 100,000—scale on the right, lines.

Deaths from COVID-19 in Poland in 2020 caused loss of 270,572 quality adjusted life years (QALYs). Men were more affected than women (160,536.11 and 865.32 per 100,000 vs. 110,035.93 and 555.67 per 100,000). On average each person who died from COVID-19 lost 6.76 QALYs (6.94 men vs. 6.52 women). Age and sex distribution of both absolute and relative values showed similar patterns as in the case of LPYLL and SPYLL (Figure 3). 38% of QALYL accounted for persons aged under 65. When considered separately both sexes, this figure was higher for men than for women (42% vs. 31%).

QALYL caused by COVID-19 morbidity was estimated for 6198,96 (16.14 per 100,000 inhabitants), of whom 1352.43 (22%) resulted from hospitalization. The overall burden of COVID-19 in Poland in 2020 was estimated for 276,762 QALYs, of which QALYs lost due to COVID-19 morbidity was accounted for 2% and QALYs lost due to COVID-19 mortality was accounted for 98%.

### 3.4. COVID-19 Burden in Relation to Burden from Other Disease Mortality

The LPYLL, SPYLL and QALYL due to all-cause mortality for both sexes in Poland in 2020 amounted to 5,728,648.46, 8069,150.65 and 3,631,618,39, respectively. When these values were taken as a point of reference, the loss of life years due to COVID-19 accounted for 8% of all-cause mortality-related LPYLL and SPYLL and 7% of all-cause mortality-related QALYL. For comparison, life years lost due to the most common causes of deaths in Poland—cardiovascular diseases and cancers—constituted 29% and 24% of all-cause mortality life years lost, respectively. Burden of COVID-19 in terms of years of life lost is proportionate to external-cause deaths and higher than the burden of diseases of the respiratory system (Table 1).

Mean LPYLL, SPYLL and QALYL due to COVID-19 among men were lower than mean all-cause mortality LPYLL, SPYLL and QALYL (Table 1). On average, each man who died of COVID-19 lost less LPYLL and SPYLL than a man who died due to neoplasms, diseases of circulatory system and external causes, and less QALYL than a man who died of neoplasms and external causes (Table 1). A woman who died of COVID-19 lost on average more LPYLL, SPYLL and QALYL than a women who died of diseases of the circulatory system and less than a woman who died of neoplasms and external causes. For both men and women, mean LPYLL, SPYLL and QALYL due to COVID-19 mortality and diseases of the respiratory system are similar (Table 1).

## 4. Discussion

Our study provides quantitative evidence of the COVID-19 burden in Poland and identifies the varying effect of COVID-19 across sexes and age groups. In absolute and relative terms, COVID-19 represents a significant loss of life years, which could potentially be avoided by optimal prevention and treatment. The revealed differences between sexes with respect to YPLL and mean YPLL reflect the fact that men died of COVID-19 not only more frequently than women, but also were more likely to die at a younger age (<65). The analysis and interpretation of COVID-19 death registry using YPLL and mean YPLL lead to a more accurate assessment of the impact of the disease on different age groups. The fact that deaths of patients younger than 65 resulted in approximately 35% of COVID-19-related LPYLL and SPYLL and 38% of QALYL would not be expected a priori based on the number of deaths in this age group alone (17% of all deaths due to COVID).

In our study, we express the burden of COVID-19 as LPYLL, SPYLL and QALYL. LPYLL was calculated using Polish life tables and therefore reflects the country-specific burden of disease, which might be useful for local public health decision makers. SPYLL was calculated applying standard reference life tables and may serve as an additional measure useful for international comparative studies and global health estimates of the burden of diseases. QALYL quantify country-specific years of life lost adjusted by age-specific utility values obtained in the Polish population and allows us to assess the impact of both COVID-19 mortality and morbidity.

Taking into consideration that country-specific information is of higher importance for local public health policymakers, LEYLL and QALYL calculations seem to be more useful for this audience than SEYLL. On the other hand, SEYLL should be recommended as the best strategy allowing the results to be potentially globally comparable, provided that the methods of calculation comply with the scientific standards currently in force. GBD 2015 methodology applied in our study meet the mentioned requirements.

Our study contributes to an understanding of the burden of COVID-19 and indicates how important our standardization of methods is used for the calculation of potential years of life lost adequately to a specific audience. Methods of calculation of SPYLL, LPYLL and QALYL presented in our study should be applied on national and international levels in order to assess the risk and potential health needs in any future epidemic conditions.

A direct comparison of our findings and findings from studies on COVID-19 burden in different countries is difficult due to methodological differences. Firstly, studies were performed at different stages of the pandemic trajectory and in different geographical areas. For instance, Quast at al. calculated YLL due to COVID-19 in the United States as of July 2020 [15], Mitra at al. analysed data from United States, Germany and Italy until 30 May 2020 [16], Ferenci et al. used Hungarian data as of 12 May 2021 in population above 50 years of age [17], Castro et al. applied Brazilian data from the 16 February 2020 to the 1 January 2021 [18], Piffare et al. analyzed the premature mortality impact of COVID-19 by calculating the amount of YLL across 81 countries as of 6 January 2021 (in 35 of the countries in this sample, coverage of the data spans at least 9 months) [19], Vieira et al. calculated YLL due to COVID-19 from week 10 to week 52 in 2020 for eight European countries (Portugal, France, Spain, Italy, The Netherlands, Germany, Sweden, UK) [20], Rommel et al. estimated YLL due to COVID-19 in Germany in 2020 [21], Ugarte et al. investigated burden of COVID-19 in 17 countries and territories across the world between January and August 2020 [22]. YPLL calculations either assumed a fixed target age to which years lost is measured [16,18], or used a life table to calculate the expected remaining time (i.e., time lost) for each death [17,19,20,21,22]. An additional limitation that poses a challenge in comparisons between countries is that the range of age groups in which deaths are grouped varies considerably from country to country. Only some studies reported YPLL per 100,000 population or per death [16,17,18,21,22], or used both reported deaths and excess deaths to account for the potential undercounting [17,18], adjusted the residual life expectancy entering the YLL calculation for pre-existing conditions [15,17] or attempted to contextualize the burden of COVID-19 performing a comparison with YLL due to other diseases [18,19,20,21]. Only in one study have disability-adjusted life years (DALY) lost to COVID-19 been estimated to account for both fatal cases and years lived with disability [21].

Some of our findings are consistent with the results of other studies. Firstly, the majority of deaths and YLL occurred among people aged above 65, justifying national policies aimed at protecting this population. Secondly, the majority of deaths and YLL have occurred in men, suggesting that sex-specific policies might be equally justified as policies based on age. Disparities in sex-specific YPLL arises from two causes: more men are dying from COVID-19 and men are dying at younger ages. Although this general pattern is present in most countries, both the size of the disparity and the importance of the two above causes varies significantly. The ratio of YPLL per 100,000 in men to YPLL per 100,000 in women spans from near 1 in Finland or Canada, to more than 2 in countries like Peru or 4 in Taiwan [19]. It was observed that highly skewed men to women YPLL rates were prevalent in low-income countries and that this imbalance resulted mostly from the death count disparities across sexes [19]. International comparisons showed that in heavily impacted highly developed countries, COVID-19 is between 25% and 50% of the YPLL rates attributable to heart conditions [19], which is in line with our findings. A German study revealed that 99.3% of the COVID-19 burden accounted for fatal cases and 0.7% for years lived with disability [21]. A similar proportion was seen in our analysis, where QALYL due to COVID-19 morbidity accounted for 2% and QALYL due to COVID-19 mortality accounted for 98%.

Our study has several limitations. Firstly, COVID-19 deaths by age and sex may be undercounted, as the numbers provided by Statistics Poland and used in our study constituted about 60% of noted mortality increase in Poland in 2020. However, to date Statistics Poland provide the most reliable data. Secondly, as in the Global Burden of Disease study, in our analysis we did not address the impact of comorbidities on the life expectancy of the deceased separately. For each deceased, years of life lost are equal with the residual life expectancy that average persons of the respective age have according to the period life tables. This approach did not consider the fact that person who died due to COVID-19 might be in a pre-existing worse health state that another person at the same age.

In Poland, 83.1% of patients with COVID-19 who died during hospitalization had at least one comorbidity [23]. However, it should be stressed that in our study LPYLL and QALYL were calculated using country-specific data on sex, age-specific life expectancy and health state utilities.

From a statistical point of view, the residual life expectancy represents the average life expectancy that can be achieved at each respective age, including healthy and ill persons. In the older population, the prevalence of multimorbidity is relatively high and “average” life expectancy and to a greater extent the “average” quality adjusted life expectancy applied in our study reflected pre-existing morbidity of those who died of COVID-19. Moreover, modelling study performed by Hanlon et al. [24] showed that adjustment for the number and type of long-term conditions did not drastically impact the YLL estimated using a standard approach: across both men and women, the number of YLL per death dropped from 14 and 12 to 11.6 and 9.4 years, respectively.

In our study we tried to calculate QALY L due to both mortality and morbidity. Due to the lack of reliable data our analysis does not consider the long-term COVID-19 effects and included only the impact of acute disease on QALYL. A deeper understanding of the late consequences in COVID-19 survivors will produce better estimates of the burden of this disease in future years.

Another limitation of our study is that a COVID-19 outbreak is still ongoing and rapidly evolving, and therefore the presented results may not reflect ongoing issues related to the pandemic. Our study presents the situation before an immunization plan against COVID-19 has been introduced and shows what happened without the availability of an effective vaccine. Currently, the epidemiologic situation is different and new problems have arisen, e.g., acceptance of COVID-19 vaccination [25]. A comparison of estimates generated at the early stages of the pandemic with data from a later phase might be important to test the utility of several measures and surveillance systems adopted and enforced to limit the spread of the virus and its burden. In this way, we can be better prepared for future pandemics.

## 5. Conclusions

Our national estimates of LPYLL, SPYLL and QALYL due to COVID-19 allowed us to fully contextualize the demographic impact of the pandemic seen in 2020 in Poland. Differential effects by sex and age indicate important heterogeneities in the mortality effects of COVID-19 and justifies policies based not only on age but also on sex. Compared with YPLL, for other diseases it was shown that mortality from COVID-19 represents a substantial burden on both the society and individuals in Poland. These findings relate to the specific conditions in 2020 and can serve as the starting point for later studies, particularly when vaccination or other strategies will alter susceptibility or severity of infection. It will be vital to continue to monitor YPLLs due to COVID-19 in absolute and relative terms to inform on clinical responses to COVID-19 and the effect of this disease on mortality from other causes. Moreover, our study validates that SPYLL is essential when the measurement is applied to compare across countries or different populations, while calculation of LPYLL and QALYL is a better measure of premature life lost for local public health decision makers.

## Figures and Tables

**Table 1:** Deaths and disease burden (LPYLL ^1^, SPYLL ^2^, QALYL ^3^) in Poland in 2020.

	All-Cause Mortality		COVID-19		Diseases of Circulatory System		Neoplasms		Diseases of Respiratory System		External Causes of Death	
	Men	Women	Men	Women	Men	Women	Men	Women	Men	Women	Men	Women
LPYLL												
absolute	3,248,501	2,480,197	250,566	185,795	865,076	770,480	694,435	702,510	173,510	128,294	384,288	102,284
%	100%	100%	8%	7%	27%	31%	21%	28%	5%	5%	12%	4%
mean	13	11	11	11	16	8	13	15	11	10	26	19
per 100,000	17,511	12,525	1351	938	4663	3891	3743	3548	935	648	2 071	517
SPYLL												
absolute	4,999,030	2,480,197	397,643	232,384	1,369,398	967,888	1,104,064	870,665	271,069	159,738	537,102	119,207
%	100%	100%	8%	8%	27%	32%	22%	28%	5%	5%	11%	4%
mean	20	13	17	14	25	10	20	19	17	13	36	23
per 100,000	26,946	15,504	2143	1174	7382	4888	5951	4397	1461	807	2 895	602
QALYL												
absolute	2,144,948	1,486,670	160,536	110,036	552,773	442,985	451,722	428,422	111,808	76,121	272,252	67,009
%	100%	100%	7%	7%	26%	30%	21%	29%	5%	5%	13%	5%
mean	9	7	7	7	7	5	8	9	7	6	18	13
per 100,000	11,562	7507	865	556	2980	2237	2435	2163	603	384	1468	338
Deaths												
absolute	249,744	227,611	23,136	16,887	83,435	95,313	55,496	46,056	16,197	12,502	14,908	5289
%	100%	100%	9%	7%	33%	42%	22%	20%	6%	5%	6%	2%
per 100,000	1346	1149	125	85	450	481	299	233	87	63	80	27

^1^ country-specific (local) potential years of life lost; ^2^ standard potential years of life lost; ^3^ quality adjusted years of life lost.

## Data Availability

Not applicable.
